# Scotland and the College

**Published:** 1921-06-25

**Authors:** 


### THE MATRONS' AND SISTERS' DEPARTMENT.

### SCOTLAND AND THE COLLEGE.

Members of the College of Nursing from all parts of the Kingdom, meeting this week in the Northern capital, have good reason for experiencing some elation of spirits. British nurses have never been conscious of any division of interests or ideals whether their training-school may happen to be situated in England, Scotland, or Ireland. And their essential unity is reinforced and brought vividly before the public in the brilliant success of their joint College, celebrated at the first annual meeting to be held in Edinburgh.

The Scottish Board has reported that the outlook of the College in Scotland grows brighter each year. Already Centres are in existence in Aberdeen, Dundee, Edinburgh, and Glasgow, and to these will shortly be added one in Ayrshire. The residential and holiday club in Drumsheugh Gardens is already an assured success, and at Glasgow the Centre has its headquarters at the Glasgow Nurses' Club, where, by the courtesy of Miss May Reid, members have the use of the rooms for post-graduate lectures and social meetings. At Aberdeen the support and generosity of Lord and Lady Cowdray in the matter of the Club are warmly appreciated; indeed, these kind friends form an additional link between the English and Scottish branches of the College. Dundee has an admirable Rest Home for Nurses, which is a great boon to the Branch.

Numerically the Scottish Branch is gaining in strength. Out of 308 applications for membership 284 were accepted last year by the Registration Board, and only three resignations have been reported. The high standard of qualification set by the College renders membership an honour and exercises a marked influence on public opinion.

It is not difficult to gauge the extent to which Scottish nurses benefit under the establishment of the College. In all particulars the Centres are on equal terms. The greater strength and importance of the English headquarters reflects good on all. Certain functions can only be exercised for all alike from one common centre, and in the issue of the *Bulletin*, in the accident and illness insurance, in the recommended scale of salaries, in the proposed national superannuation scheme, in the Loan Fund, and in the Nation's Fund for Nurses the interests of British nurses are completely united. Individual enterprises, such as the provision of scholarships, the promotion of post-graduate courses, the formation of appointments and information boards, and other local schemes are calculated to arise in every quarter as and when required. Noting with great pleasure the Carnegie grant of £500 towards a professional library for nurses at the new Cowdray Club in Cavendish Square, we cannot forbear expressing the hope that Scots members will not let London get ahead of them in this particular. It must be the aim to secure for Edinburgh whatever is secured for London, so far, at any rate, as professional advantages are concerned, and foremost among these we place the opportunity for study and encouragement for research.

It is by no means probable that Scotland will be content only to follow on. We predict that Scots nurses will be found fertile in invention, strong in powers of developing their organisation, and wide-minded in promoting the success of the College in all its branches. We confidently look for them to initiate as well as loyally to carry out plans which already spell success. Working together shoulder to shoulder during the war, Scots and English nurses learned to understand each other's quality. Without depreciating nurses of other climes, it may be suggested that the Scotswoman is dowered at birth with an instinct, faculty, or call it what you will, which, rightly directed, in the wards, as in the home, works for good management. Under its decentralised system, allowing full play for national characteristics to develop freely, the College of Nursing will owe much in the future, as in the past, to the organising ability of its members over the Border.

Whether the annual meeting has surprises in store for the assembled members or no, the record of work accomplished during the sixth year of its existence will surely render it memorable.

